# Skill transfer from symmetric and asymmetric bimanual training using a robotic system to single limb performance

**DOI:** 10.1186/1743-0003-9-43

**Published:** 2012-07-17

**Authors:** Matic Trlep, Matjaž Mihelj, Marko Munih

**Affiliations:** 1 Faculty of Electrical Engineering, University of Ljubljana, Trzaska 25, 1000 Ljubljana, Slovenia

## Abstract

**Background:**

Humans are capable of fast adaptation to new unknown dynamics that affect their movements. Such motor learning is also believed to be an important part of motor rehabilitation. Bimanual training can improve post-stroke rehabilitation outcome and is associated with interlimb coordination between both limbs. Some studies indicate partial transfer of skills among limbs of healthy individuals. Another aspect of bimanual training is the (a)symmetry of bimanual movements and how these affect motor learning and possibly post-stroke rehabilitation.

**Methods:**

A novel bimanual 2-DOF robotic system was used for both bimanual and unimanual reaching movements. 35 young healthy adults participated in the study. They were divided into 5 test groups that performed movements under different conditions (bimanual or unimanual movements and symmetric or asymmetric bimanual arm loads). The subjects performed a simple tracking exercise with the bimanual system. The exercise was developed to stimulate motor learning by applying a velocity-dependent disturbance torque to the handlebar. Each subject performed 255 trials divided into three phases: baseline without disturbance torque, training phase with disturbance torque and evaluation phase with disturbance torque.

**Results:**

Performance was assessed with the maximal values of rotation errors of the handlebar. After exposure to disturbance torque, the errors decreased for both unimanual and bimanual training. Errors in unimanual evaluation following the bimanual training phase were not significantly different from errors in unimanual evaluation following unimanual training. There was no difference in performance following symmetric or asymmetric training. Changing the arm force symmetry during bimanual movements from asymmetric to symmetric had little influence on performance.

**Conclusions:**

Subjects could adapt to an unknown disturbance torque that was changing the dynamics of the movements. The learning effect was present during both unimanual and bimanual training. Transfer of learned skills from bimanual training to unimanual movements was also observed, as bimanual training also improved single limb performance with the dominant arm. Changes of force symmetry did not have an effect on motor learning. As motor learning is believed to be an important mechanism of rehabilitation, our findings could be tested for future post-stroke rehabilitation systems.

## Introduction

Humans have the important ability to adapt to new unknown dynamics affecting their movements. When an unknown velocity-dependent force field is applied to the arm during reaching movements, initial perturbations are large and then decrease over time [1,2]. Such motor learning is believed to be an important mechanism of rehabilitation following stroke [3].

Previous post-stroke studies have demonstrated that bimanual training improves dexterity, grip strength and functional ability of the paretic limb [4,5]. It has been suggested that, following bimanual training, the contralesional (undamaged) brain hemisphere might provide a template of appropriate neural responses for a restored neural network. Changes in the contralesional hemisphere of some patients were reported after bimanual training [6]. Simultaneous activation of both arms might facilitate motor learning.

The benefits of bimanual training are often associated with interlimb coordination between both limbs [7]. Although some studies indicate partial transfer of skills from shared bimanual tasks to a single limb [8], the amount of beneficial transfer is still uncertain even in healthy nervous systems. The effects of bimanual transfer could prove to be beneficial for people with hemiparesis since the less affected arm could potentially “instruct” the impaired arm on how to move. Since bimanual control deficits have scarcely been systematically investigated in the context of stroke, many uncertainties remain about the adequate prescription and the true value of bimanual movement training [7].

Other studies involving bimanual coordination such as temporal characteristics of bimanual targeted-reaching tasks [9] and synchronous circle drawing tasks [10] found that coupling of movements did not provide a benefit to the impaired limb. This variation in results involving bimanual transfer with different tasks can not be explained because the underlying functional mechanisms are not yet completely understood and more research has to be done in this field.

In healthy individuals, the transfer of skills between limbs varies depending on the direction of the transfer (towards the dominant or nondominant limb) and on the type of skill [11,12]. It has been also suggested that the transfer can be excluded by introducing the force field gradually [13]. Some experiments have looked at bimanual training with transfer to single-limb performance (e.g. [8,14]). This is quite different from executing a task with only one limb and then another, because bimanual tasks stimulate coupling of the limbs while this would not be expected in unimanual practice. Research of connections between unimanual and bimanual training has shown that rehabilitation may be facilitated by bimanual motor practice, but is likely to require further unimanual training to maximize motor recovery [15].

Several studies have shown and explained how the motor system effectively plans and accommodates for different inertial loads [16,17]. According to these studies the movements are performed in a feed-forward manner based on an internal model of the task dynamics [16]. However, if the dynamics change and the central program fails to produce the desired trajectory, the errors are compensated by sensory feedback signals compared to the kinematic reference plan. The internal model of the task dynamics then also changes according to the feedback interventions. Further research has shown that bimanual movements with inertial loading on unaffected limb may increase interlimb coupling and can be used as an useful addition to bimanual training in chronic hemiplegia for some individuals [18]. These studies researched the interlimb coupling, but do not give any information about the asymmetric load distribution effects on motor learning during bimanual task execution.

In recent years, the use of robotic systems as guidance and evaluation devices has been introduced in post-stroke rehabilitation. Several studies have examined the effects of robotics on paretic arm function recovery in rehabilitation of stroke patients [19-23]. Various robotic devices have been developed to promote bimanual training of upper extremities [23-25]. These studies showed that combined unimanual and bimanual robotic training has advantages compared to conventional therapy only [24,26].

In our previous study, we investigated the effects of adaptive bimanual robotic training on chronic hemiparetic subjects [23]. Hemiparetic subjects recovering from stroke have limited functions of the impaired arm compared to the less affected arm. To enable the subjects to complete the bimanual exercises in spite of this, the less affected arm needed to apply greater forces in order to help the impaired arm. During bimanual training, the force ratio of both arms changed according to the subjects performance. Bimanual training resulted in improved task specific performance, both in bimanual and in unimanual reaching movements. These results indicated some connections between bimanual and unimanual movements.

In the present study, our goal is to determine the degree to which the human motor system can adapt to a velocity-depended force field during shared bimanual reaching task. One goal is to investigate the transfer of motor patterns learned during bimanual training towards unimanual movements using the dominant arm. Furthermore, the effect of different arm load distributions on the transfer of motor learning during bimanual training will be addressed, as it could explain the role of adaptive bimanual training in rehabilitation.

## Methods

### Hardware

Our previous research was focused on upper extremity rehabilitation of hemiparetic subjects performing reaching exercises with a bimanual robotic system [23]. Experiments with the bimanual robotic system showed that devices for bimanual training should be more simple and adaptable to the individual needs and abilities of every individual.

**Figure 1:**
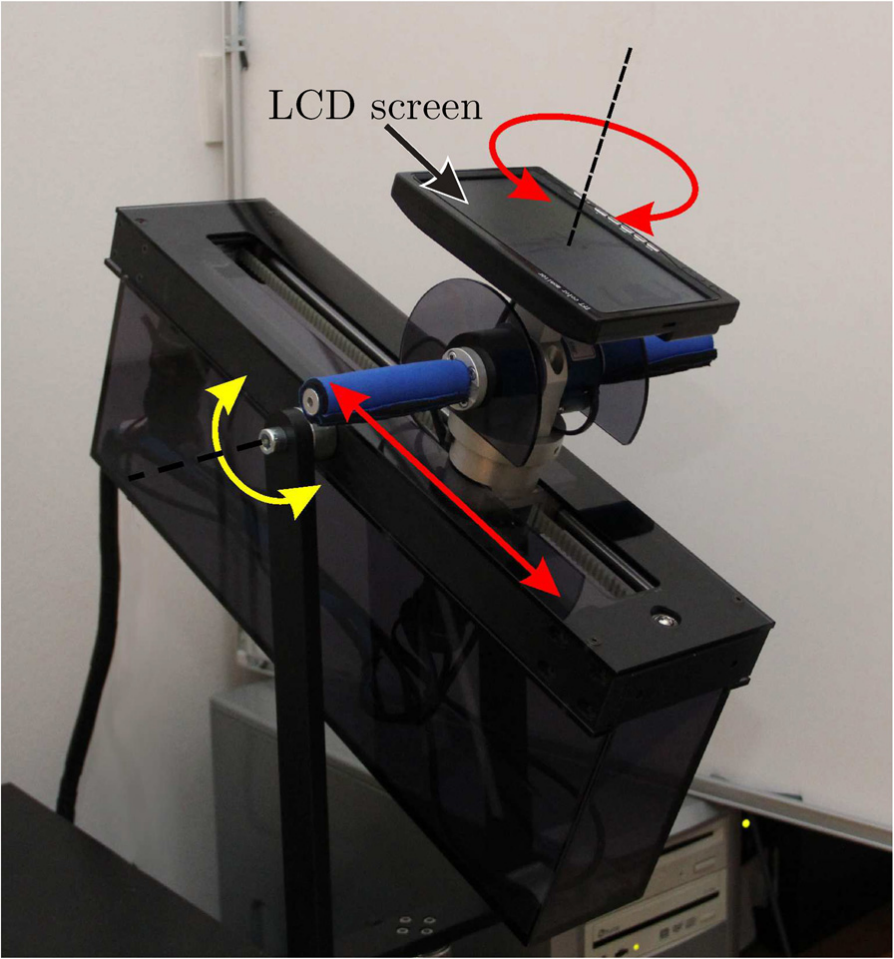
**Bimanual training system.** The two active degrees of freedom are indicated by the red arrows, while the yellow arrow indicates the tilt of the system.

**Figure 2:**
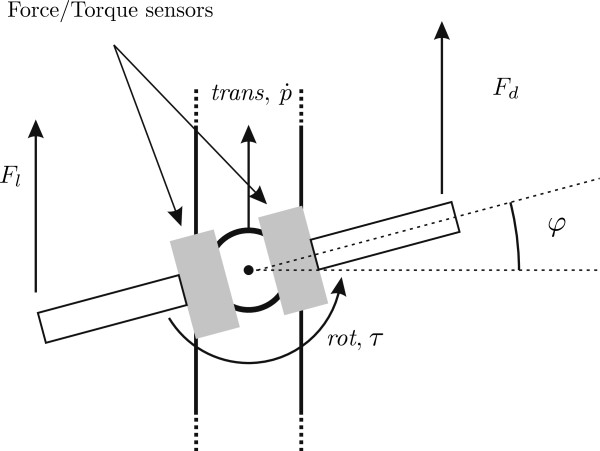
**Top view of the bimanual handlebar.** Forces of the left *F*_*l *_and right arm *F*_*r*_, the rotation *φ *and translation velocity $\dot{p}$ of the handlebar are presented. The translation (*trans*) and rotation axis (*rot*) are presented.

The proposed bimanual training system has two active degrees of freedom (DOF) indicated by the red arrows in Figure 1. The translation of the robot end-effector is possible in one direction and the end-effector can rotate around an axis perpendicular to the direction of the translation. For active feedback, a Maxon DC motor RE40 with a gearhead GP42C (reduction rate 26:1) is used to control the translation of the end-effector while a Maxon DC motor RE30 with a gearhead GP32C (reduction rate 23:1) is used to control the rotation of the end-effector. The maximal translational force is limited to 200 N. The maximal continuous rotation torque is 2 Nm while the maximal temporary torque is limited to 5 Nm. Using an encoder with 500 counts per turn, the positional resolution of the translatory movement is 0.01 mm. The resolution of the handlebar rotation is 0.01 degrees using a 512-counts-per-turn encoder. The DC motor is connected to the handle with a tooth belt that moves the handle along linear guides. The range of the translation is 35 cm while the rotation is physically limited to ± 45° due to safety reasons. For safety during the exercises the operator must hold a dead man’s switch at all times. An extra passive rotation enables the pitch of the device to be adjusted (the yellow arrow). The bimanual handlebars (Figure 2), with each handle 11 cm long, are mounted on the end-effector and independently measure forces generated by each arm using two 6-DOF force and torque sensors (50M31, JR3 Inc.). The force sensor has a range of ± 100 N for its *x* and *y* axes, and a range of ± 200 N for its *z* axes. The resolution of the sensor is 0.04 N (*x* and *y* axes) and 0.08 N (*z* axes). The data were collected at a sampling frequency of 2500 Hz. The handlebar turns like a steering wheel and can actively resist the subject’s steering. A 7-inch LCD screen with a resolution of 800 × 480 pixels is mounted on the top of the handlebar to enable visual representation of the virtual task (Figure 1). The width of one pixel is equivalent to a translatory movement of 0.2 mm. The user’s hands are not fixed to the handlebar, since healthy subjects do not need any support (fixation could be considered for patients’ paretic arm).

### Training exercises

Bimanual repetitive reaching training has been reported to be efficacious in promoting upper-extremity recovery in chronic stroke [23,27]. The potential of bimanual training inspired us to design a simple bimanual reaching exercise where the limbs are constrained to act as a single unit by virtue of mutual coupling. In such a system the, arms are coupled in a way that allows one arm to support the other if needed. This is one possible approach for post stroke rehabilitation and was shown to be effective in our previous study [23]. In the present study, we want to look closely at the principles involved in this kind of bimanual training.

**Figure 3:**
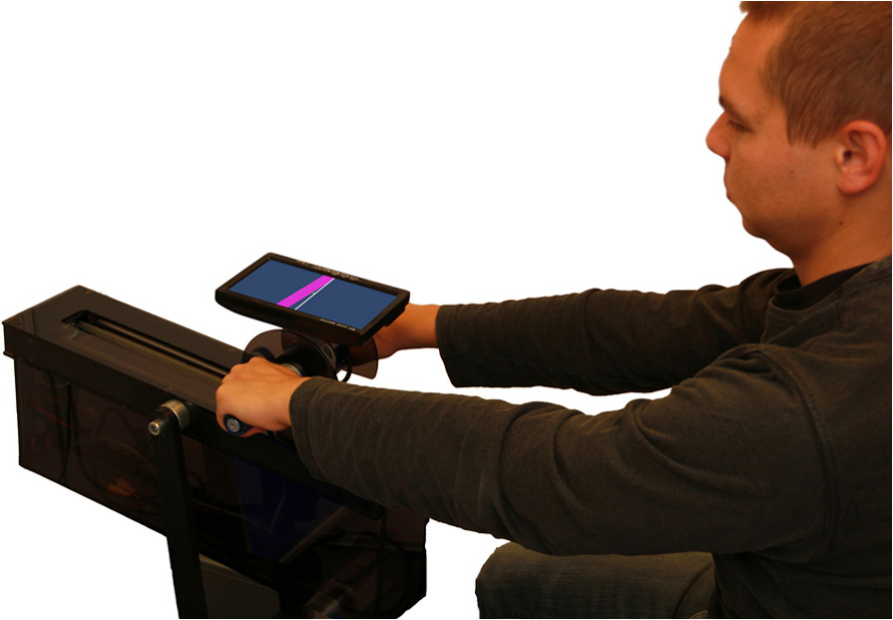
**Subject during training.** Subject moves the handlebar with one or both arms. The virtual tracking exercise is displayed on the screen mounted on the handlebar.

**Figure 4:**
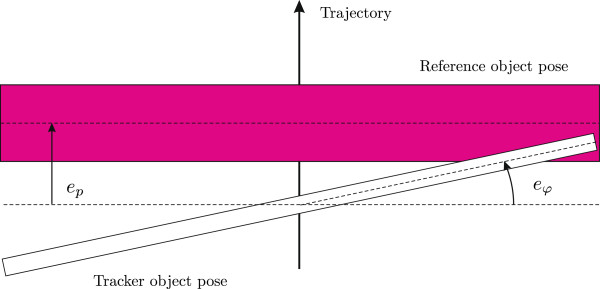
**Virtual tracking exercise.** The reference object moves on a predefined trajectory and the subject must follow it by moving the tracker object (bimanual handlebar). *e*_*φ *_is the rotation error and *e*_*p *_is the tracking error.

**Figure 5:**
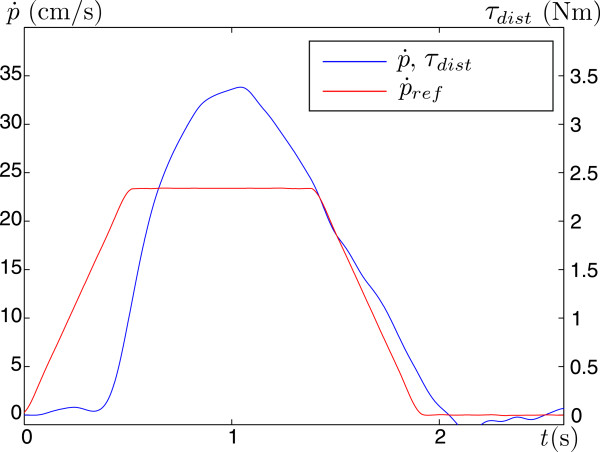
**Velocity of the handlebar**$\dot{p}$**, reference velocity**${\dot{p}}_{\mathrm{ref}}$**, and disturbance torque *τ***_***dist***_**.** The predefined reference velocity (trapezoidal velocity profile) is in every trial the same. The translational velocity also corresponds to the disturbance torque (Equation 2).

The training exercise was designed to be performed in the sagittal plane in front of the subject who is sitting behind the bimanual device (Figure 3). The subject is seated so, that he/she can comfortably reach the furthest point of the translation movement by moving the handlebar according to the virtual task on the screen (Figure 4). The system’s tilt is set at approximately 15 degrees. A *reference object* (horizontal pink bar) displayed on the screen moves along a predefined trajectory independent of the pose of the bimanual handlebar (and the screen). The trajectory is determined by a trapezoidal velocity profile (also see Figure 5 in the Results section for a time-dependent profile of the trajectory), with equal positive and negative acceleration that both lasted 0.5 seconds. The peak velocity was set at 24 cm/s and lasted for 0.6 seconds during the forward movement. In order to simplify the task, the reference object orientation is kept horizontal. The subject is required to track the reference object pose by moving the robot end-effector indicated with a *tracker object* also displayed on the screen (white line) as shown in Figure 4. In the bimanual mode, the user holds the handlebar with both hands and must coordinate them to keep the tracker object orientation constant. The unimanual mode requires the use of only the dominant arm to manipulate the handlebar.

The task was designed to stimulate activation of muscles by applying resistance to the movements. The resistance stimulates sensory-motor system activation in the stimulated direction (forward) and is not used in the return movement. Resistance during unimanual trials is set to 50% of the resistance during bimanual trials so that the required forces of each arm are comparable. In general, bimanual movements are not necessarily done symmetrically, as one arm can participate more in the movement than the other arm. In order to assess the effect of applying different forces with each arm on motor learning, some subjects had to apply forces asymmetrically: one arm had to apply greater force to the handlebar to keep it balanced. *The arm force ratio* is therefore defined as the predefined ratio of left and right arm forces required to keep the handlebar balanced.

### Control strategies

An admittance controller was chosen to control the bimanual training system. The robot is controlled by applying force to its end-effector. As the system is bimanual, the scaled forces of both arms are summed to produce the control force

(1) $$F_{c}=K_{l}F_{l}+K_{r}F_{r}.$$

*F*_*l*_ and *F*_*r*_ are scalar values of the left and right arm forces in the direction of the translation axes (Figure 2). Gains *K*_*l*_ and *K*_*r*_ ensure the desired arm force ratio between arms. If the desired mode is *symmetric* where both arms participate equally, then *K*_*l *_=* K*_*r *_= 1 (arm force ratio of 50*%*:50*%*). During the *asymmetric mode*, the controller requires the forces of one arm to be greater than those of the other. To ensure this, forces of one arm are scaled down (a greater force is needed for the same effect), while the forces of the other arm are scaled up (a smaller force is needed for the same effect). *K*_*l *_= 0.5 and *K*_*r *_= 1.5 when the left arm should apply greater forces to the combined movement (*asymmetric mode* – arm force ratio of 75*%*:25*%*), while *K*_*l *_= 1.5 and *K*_*r *_= 0.5 when the right arm should apply greater forces.

During some trials, the motor of the top rotation applied a *disturbance torque* to the handlebar. The disturbance torque *τ*_*d *_is presented as a torque that tries to rotate the handlebar in the direction of the dominant hand. *τ*_*d*_ is defined as

(2) $$\tau_{d}=\left\{\begin{array}{lcc} B\dot{p} & \text{for} & \dot{p}>0,\\ 0 & \text{for} & \dot{p}\leq 0, \end{array}\right)$$

where $\dot{p}$ is the forward velocity of the handlebar (Figure 2) and *B* is a constant gain defined as *B *= 10 Ns. For trials without the disturbance torque, *τ*_*d *_is defined as *τ*_*d *_= 0 Nm.

Similarly to the control force in Equation 1, a control torque is determined from the left arm torque *τ*_*l*_ and right arm torque *τ*_*r*_ with addition of the disturbance torque *τ*_*d *_as

(3) $$\tau_{c}=K_{l}\tau_{l}-K_{r}\tau_{r}+\tau_{d}.$$

The admittance controller used to control the 2-DOF mechanism is implemented via a second order admittance model

(4) $$\begin{array}{ll} F_{c}=m{\ddot{p}}_{r}+b_{p}{\dot{p}}_{r}, & \; \end{array}$$

(5) $$\begin{array}{ll} \tau_{c}=I{\ddot{\varphi }}_{r}+b_{\varphi }{\dot{\varphi }}_{r}, & \; \end{array}$$

where *m* represents the virtual mass (*m *= 3.5 kg), *I* represents the virtual moment of inertia (*I *= 0.1kgm^2^), and *b*_*p*_ and *b*_*φ*_ represent the virtual viscous damping of the system (*b*_*p *_= 50 Ns/m and *b*_*φ *_= 0.7 Nms/rad). In order to reduce the resistance of the robot during unimanual mode, the viscous damping during unimanual trials is at 50% of the resistance during bimanual trials (*b*_*p *_= 25 Ns/m and *b*_*φ *_= 0.35 Nms/rad). This ensures that the forces applied by the arms in both types of movement are similar. The reference positions *p*_*r*_, *φ*_*r*_ and velocities ${\dot{p}}_{r}$, ${\dot{\varphi }}_{r}$ are computed from differential equations (4) and (5) and then used in the robot proportional-derivative (PD) position controller not presented here.

The controller for the system was designed as a Matlab/Simulink model and implemented on an xPC Target PC with a control loop rate of 2500 Hz.

### Experimental protocol

Before the study began, informed consent was obtained from the subjects and ethical approval was obtained from the National Medical Ethics Committee of the Republic of Slovenia. 35 healthy young adults without any motor disabilities participated in the study. The subjects were instructed to track the pose of the reference object on the screen that followed a predefined trajectory with a trapezoidal velocity profile and constant orientation by moving the handlebar (Figure 3). Each trial started at the same starting point. A countdown on the screen and an audio signal indicated the start of each trial – the reference object started moving in a forward direction. The movement was 30 cm long and the desired movement duration indicated by the reference object was 1.6 seconds. After the endpoint was reached, the target remained in place for 1 second before returning to the starting point. After 4 seconds, the next trial started. Subjects were instructed to make smooth movements in the forward direction, matching the movement of the reference object.

Subjects were divided into 5 test groups, each consisting of 7 subjects. General characteristics of the groups are presented in Table 1. Exercise conditions for each group varied in the type of the movements (*unimanual* or *bimanual*) and in the arm force ratio – *symmetric* (50*%*:50*%*) or *asymmetric* (75*%*:25*%*, the nondominant limb applied larger forces). Conditions for all 5 groups are summarized in Table 2.

**Table 1:** Testing group characteristics

**Group**	**Age (years *± *std)**	**Gender**		**Handedness**	
		**Male**	**Female**	**Right**	**Left**
BS	27.5±1.4	7	0	6	1
BA	25.4±2.4	7	0	6	1
US	27.6±3.2	6	1	7	0
UA	27.3±4.0	6	1	7	0
U	28.8±3.2	6	1	5	2

**Table 2:** Testing group conditions at different phases

**Group**	**Baseline**	**Training**	**Evaluation**
BS	Bimanual Symmetric	Bimanual Symmetric	Bimanual Symmetric
BA	Bimanual Asymmetric	Bimanual Asymmetric	Bimanual Asymmetric
US	Unimanual	Bimanual Symmetric	Unimanual
UA	Unimanual	Bimanual Asymmetric	Unimanual
U	Unimanual	Unimanual	Unimanual

Each subject performed one session of 255 trials that followed a three-part structure with different trial conditions: 

1 **Baseline** – 85 trials without any outside disturbances and with the arm force ratio as defined for each group (see Table 2). The disturbance torque was pseudo-randomly unexpectedly activated. If performed bimanually, the arm force ratio also pseudo-randomly unexpectedly changed. Both unexpected changes happened about once in 6 trials.

2 **Training** – 85 trials with active disturbance torque with the arm force ratio as defined for each group.

3 **Evaluation** – 85 trials with the presence of the disturbance torque and with the arm force ratio as defined for each group. The disturbance torque was pseudo-randomly unexpectedly removed (catch trials). If performed bimanually the arm force ratio also pseudo-randomly unexpectedly changed while the disturbance torque still influenced the movement. Both unexpected changes happened about once in 6 trials.

The *baseline phase* allowed the subjects to become familiar with the system and to establish a baseline pattern. The effect of disturbance torque on non-learned movements was investigated during trials with unexpected activation of disturbance torque for a single trial. In some trials, if performed bimanually, the arm force ratio randomly changed to investigate the influence of different arm force ratios in the combined bimanual movement. The disturbance torque was not active in these trials, as only one of the two changes occurred during the same trial. After the baseline phase, the next 85 trials represented the *training phase*, during which the subjects were exposed to the disturbance torque in all trials. During the *evaluation phase* (last 85 trials), the disturbance torque was still active with intermittent random removal of the disturbance torque to determine the after-effects of motor learning. For groups BS and BA also the arm force ratio changed back to value presented at the baseline phase in a few random trials. The disturbance torque was active during these trials.

Each session was divided into three parts (baseline, training and evaluation). Each part was also divided into different *stages*, summarized in Table 3. Baseline phase trials (*B*) include all trials during the baseline phase at the given arm force ratio with disturbance torque not active. The trials with unexpected disturbance torque activation are represented by the baseline catch trials (*Bc*). The start of the training (evaluation) phase (*Ts* and *Es*) is presented as the first 10 movements of the training (evaluation) phase. The end of the training (evaluation) phase (*Te* and *Ee*) is presented as the last 10 movements of the training (evaluation) phase. The trials with unexpected disturbance torque deactivation during evaluation phase are represented by the evaluation catch trials (*Ec*). The baseline ratio (*Br*) represents those trials during baseline phase where the arm force ratio unexpectedly changed while the disturbance torque was not active. Evaluation ratio (*Er*) represents those trials during evaluation phase where the arm force ratio unexpectedly changed while the disturbance torque was still active (normal condition for evaluation phase).

**Table 3:** Stages of each session

**Stage**	**Description**
*B*	Baseline: trials during baseline phase without disturbance
*Bc*	Baseline catch: trials with unexpected activation of disturbance torque during baseline phase
*Ts*	Training start: first 10 trials of the training phase – with disturbance torque
*Te*	Training end: last 10 trials of the training phase – with disturbance torque
*Es*	Evaluation start: first 10 trials of the evaluation phase – with disturbance torque
*Ee*	Evaluation end: last 10 trials of the evaluation phase – with disturbance torque
*Ec*	Evaluation catch: trials with unexpected removal of disturbance torque during evaluation phase
*Br*	Baseline ratio: unexpected arm force ratio change during the baseline phase without disturbance torque (50*%*:50*%*⇔75*%*:25*%*)
*Er*	Evaluation ratio: unexpected arm force ratio change during the evaluation phase with disturbance torque (50*%*:50*%*⇔75*%*:25*%*)

The pose of the reference object and the pose of the bimanual handlebar were collected during all trials. The main parameter for assessment of performance was the *rotation error e*_*φ*_ (see Figure 5) defined as the difference between the reference rotation and the rotation of the handlebar, since the disturbance torque directly influences the rotation of the handlebar. To evaluate the subjects’ performance the maximal rotation error for each trial was determined. In addition, the RMS value of errors during the whole trial was also analyzed. As the errors are not constant during the movement, we also analyzed the RMS errors at the time interval 10% – 60% of a single trial duration, where the errors tend to be the greatest. These results are not presented in the paper, but in both cases the results show similar patterns as the presented maximal errors. The *translational tracking error* was evaluated as the maximal deviation of the handlebar position from the reference position. Statistical significance was evaluated as follows: If two groups performed the same exercise and results were compared between groups, a t-test was used (or a Mann-Whitney U test in case of violations of normality). When a single group performed two different exercises and results were compared between exercises, a paired t-test was used (or a paired sample Wilcoxon signed rank test in case of violations of normality). The threshold for significance was set at *p *= 0.05.

## Results

In each trial, the reference moved along a predefined trajectory with a trapezoidal velocity profile shown in Figure 5. The subject followed this movement with the handlebar. One typical time-dependent velocity profile of the handlebar is presented in Figure 5. The disturbance torque *τ*_*dist *_corresponds to the velocity profile of the handlebar as determined by the Equation (2).

**Figure 6:**
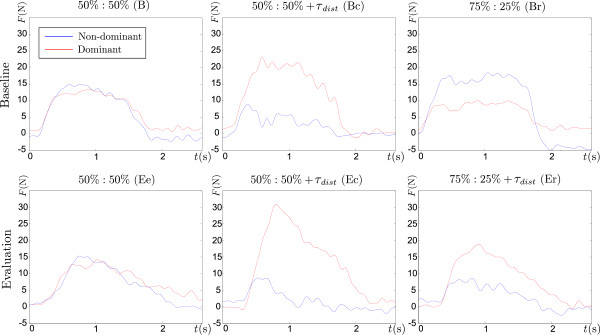
**Forces of dominant and non-dominant arms.** Forces applied at the handlebar of a group BS subject at different session conditions at the baseline and evaluation phase.

The system allows the collection of various kinematic and kinetic variables such as forces of both arms applied to the handlebar, the position and rotation of the handlebar, and velocity of the handlebar. Forces of the dominant and non-dominant arms of one subject are presented in Figure 6. The presented forces represent a typical control approach. The trials were collected from a subject in group BS at typical stages after the adaptation phase. Subjects in different groups applied similar forces.

**Figure 7:**
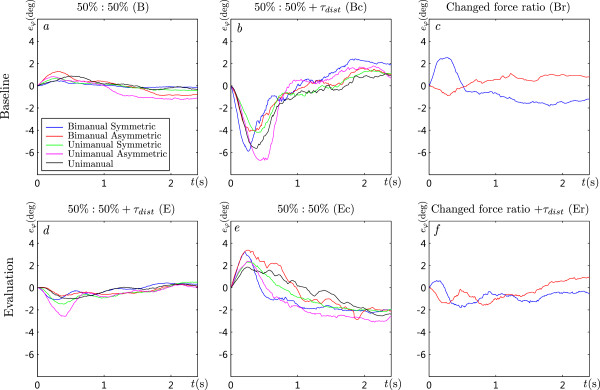
**Median rotational errors.** Time profiles of median rotational errors for all groups at different session conditions at the baseline and evaluation phases.

The time-dependent values of the median rotational errors for all five groups are presented in Figure 7. The rotational errors for all groups during the baseline phase are small (*a*). The error increases at the start of catch trials (*b*), while the error decreases in the second part of the movement. Similar, but inverted time-dependent profiles can be noticed during the evaluation phase (*d* and *e*). The error in group BS increases at the start of the movement with changed arm force ratio, while the error does not change much for group BA (*c* and *f *).

**Figure 8:**
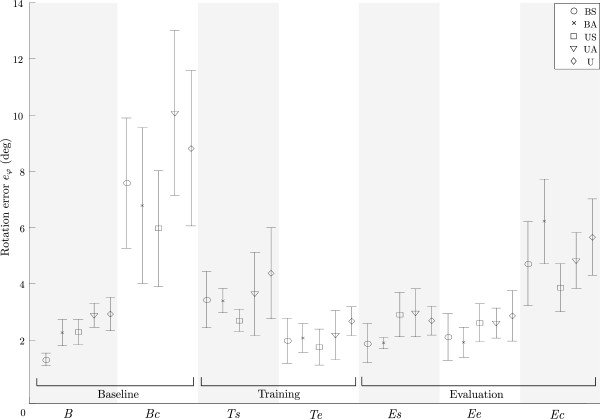
**Rotation errors for all test groups.** The rotation errors *e*_*φ *_for all groups (BS – U) are different phases: baseline movements without disturbance (*B*); catch trials during baseline phase (*Bc*); start of the training phase (*Ts*); end of the training phase (*Te*); start of the evaluation phase (*Es*), end of the evaluation phase (*Ee*); catch trials during evaluation phase (*Ec*).

The mean values of the maximal errors for all subjects in a particular test group (Table 2) are shown in Figure 8 with the standard deviation to assess the spread of data among subjects of the same group. Each session is divided into three phases and further into different stages (Table 2) presented on the horizontal axes.

Each session started with the baseline phase when the disturbance torque was not active (*B*). During this phase, the smallest error was for group BS, which performed this phase bimanually with symmetric arm force ratio. During the baseline trials with unexpected activation of disturbance torque (*Bc*), the errors for all groups significantly increased (*p *< 0.001). At the start of the training phase (*Ts*), the disturbance torque was activated and towards the end of the training phase (*Te*), the errors decreased for all groups. Significant changes can be noticed during bimanual training following bimanual baseline phase (*p *< 0.001, groups BS and BA), bimanual training following the unimanual baseline phase (*p *< 0.001, groups US and UA), and also during unimanual training (*p *= 0.020, group U). The training phase was followed by the evaluation phase with active disturbance torque that was randomly deactivated. For groups US and UA, who switched from bimanual training to unimanual evaluation, the error significantly increased (*p *< 0.001), but it did not significantly change during the evaluation phase (*Ee*). For groups BS, BA and U, there is no significant change from the end of training phase (*Te*) to the start of evaluation phase(*Es*) and end of evaluation phase (*Ee*). When the disturbance torque was suddenly removed (*Ec*), errors in all groups significantly increased compared to the end of evaluation phase (*p *< 0.05 for all groups).

**Figure 9:**
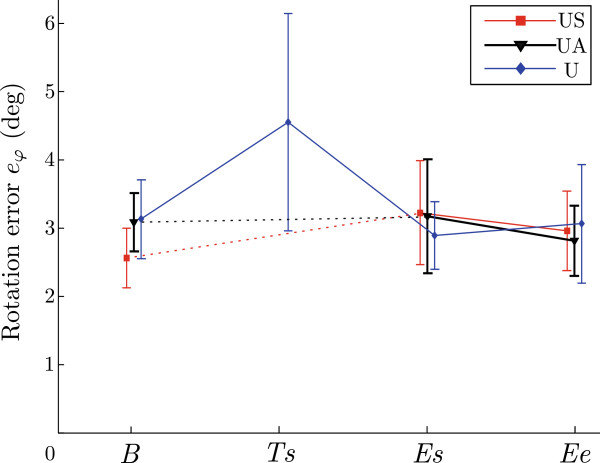
**Effects of bimanual training on unimanual performance.** Rotation errors *e*_*φ *_during unimanual baseline (*B*), start of unimanual training (*Ts* – only group U), start of unimanual evaluation phase (*Es*), end of unimanual evaluation phase (*Ee*) are shown for test groups US, UA and U.

One of the goals of the study was to investigate the effects of bimanual training on unimanual performance. The mean maximal errors of different unimanual trials for groups US, UA and U are presented in Figure 9 with the corresponding standard deviations for each group. For a detailed explanation of different phases (*B – Er*), see Table 2. The errors for these three groups during unimanual baseline phase (*B*) are presented. The errors do not show any significant difference between the groups. Next, the mean maximal error during the start of training phase (*Ts*) is presented for group U, which is the only group performing unimanual training. Errors during the start of the evaluation phase (*Es*) performed by all three groups unimanually range from 2.9° to 3.2°. These trials were all performed with a single arm with the presence of disturbance torque. To assess the effects of bimanual training on unimanual performance, we compared the start of unimanual training of group U and the start of unimanual evaluation of groups US and UA following bimanual training – both cases present the first stage of unimanual trials with disturbance torque for that group. The mean maximal error of the group U during the start of training was 4.5°, which is significantly larger than mean maximal errors of groups US and UA during the start of evaluation phase (both 3.2°, with *p *= 0.044 and *p *= 0.041, respectively). Three groups US, UA and U finished their sessions with unimanual evaluation phase (*Ee*) and the corresponding maximal errors are also presented and there is no significant difference between the groups.

**Figure 10:**
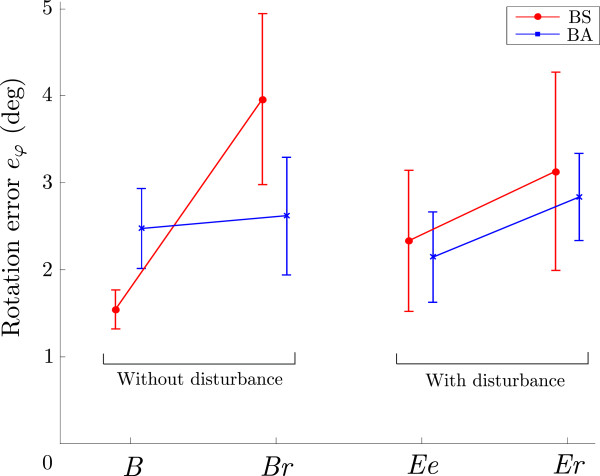
**Effects of random ratio change during bimanual trials.** First the errors during baseline phase with appropriate force ratio (*B*) and baseline phase with sudden changes to the ratio (*Br*) are presented for groups BS and BA. The right side shows the errors during evaluation phase:“normal” ratio (*Ee*) and suddenly changed ratio (*Er*).

As we also investigated the effects of different arm force ratios to the combined bimanual movement, Figure 10 shows the effects of sudden changes in arm force ratio. First, the rotation errors during the bimanual baseline phase with normal arm force ratio and with suddenly changed ratio are presented. The mean maximal errors for groups BS and BA during the baseline phase are 1.5° and 2.4°(*B*), and the mean errors when the arm force ratio changed (*Br*) are 3.9° and 2.6°. In group BS, arm force ratio changed from symmetric to asymmetric while the opposite change happened in group BA. The errors in group BS significantly increased (*p *< 0.001) while the errors in group BA did not change significantly. Similar results are shown for the evaluation phase. Errors at the end of the evaluation phase with the disturbance torque (*Ee*) are presented first (2.3° and 2.1°) followed by the errors when the arm ratio suddenly changed (*Er*) while the disturbance torque was still active (3.1° and 2.8°). A slight and statistically significant increase of errors can be noticed for group BS (*p *= 0.023) and group BA (*p *= 0.017).

**Figure 11:**
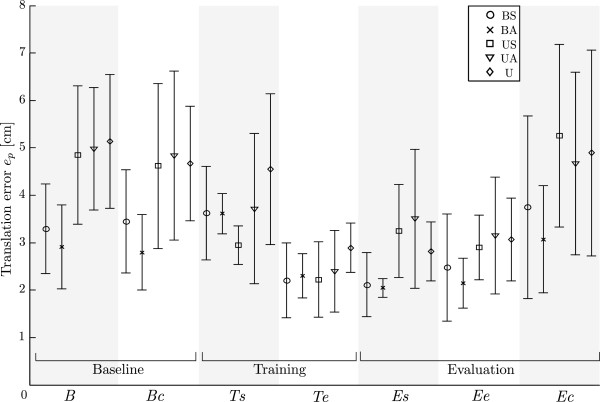
**Tracking errors for all test groups.** Tracking errors *e*_*p*_for all groups (BS – U) are presented: baseline movements without disturbance (*B*); catch trials during baseline phase (*Bc*); start of the training phase (*Ts*); end of the training phase (*Te*); start of the evaluation phase (*Es*), end of the evaluation phase (*Ee*); catch trials during evaluation phase (*Ec*).

Another parameter for task performance assessment are tracking errors *e*_*p*_ (see Figure 5). The mean maximal tracking errors for subjects at each period of the session (*B* – *Ee*) are presented in Figure 11. The introduction of disturbance torque at catch trials during baseline phase did not significantly affect the tracking errors (*B* and *Bc*) – during the bimanual baseline the the mean error was approximately 3.0 cm and about 5.1 cm during unimanual baseline. Thus, groups performing baseline movements using both arms (groups BS and BA) had significantly lower tracking errors (*p *< 0.001) compared to groups performing unimanual movements (groups US, UA and U). During bimanual and unimanual training tracking errors significantly decreased (*p *< 0.05 for all groups). Unlike in the baseline phase, tracking errors significantly increase during catch trials of the evaluation phase (*Ee* compared to *Br* with *p *< 0.001) and reach similar values as in the baseline phase. For groups BS, BA and U errors do not change significantly during the evaluation phase, while for groups US and UA (switch from bimanual training to unimanual evaluation) errors significantly increase (*p *< 0.001), but are still significantly smaller than during the baseline (*p *= 0.009).

## Discussion

The paper presents the development and validation of a novel system for both unimanual and bimanual training and the effects of bimanual training on single limb performance. The study with 35 healthy subjects showed the ability of humans to adapt to new dynamics of the environment both during bimanual and unimanual movements. A transfer of motor skills from bimanual training to unimanual performance is also observed. These results suggest that similar bimanual techniques might also be beneficial for post-stroke rehabilitation.

There are many possible control strategies to move the handle during bimanual movements. When analyzing the forces in Figure 6 during bimanual movements, it is seen that the users mostly control the handle symmetrically, as it is the most intuitive method. Other control methods are also possible, but difficult to apply, because applying a certain torque also influences the corresponding force. We believe that this is a consequence of four control variables (two forces and two torques) controlling two degrees of freedom of the handlebar. The median rotational errors during the trials by all groups are presented in Figure 7. The errors during the baseline phase are small, as the subjects get familiar with the system. During the catch trials at the baseline phase, the error at the first part of the movement significantly increases in the negative direction, while in the second part the subject’s feedback control reduces the error. The median errors during the evaluation phase are small, as the subjects learned the changed dynamics of the system. The errors at the evaluation catch trials increased in the positive direction, similarly to the baseline catch trials. The error also increased at the start of the movement when the arm force ratio suddenly changed from symmetric to asymmetric (Figure 7c and *f *, group BS), while the opposite changes did not affect the errors (Figure 7c and *f *, group BA).

The profile of the rotational error was investigated in Figure 7, where it is seen that the maximum errors occurred in the first 0.5 seconds of the trial – during the subconscious feedforward component of the torque compensation strategy applied by subjects. During the rest of the trial the subject’s feedback controller significantly reduced the rotation error on average. The method using maximum absolute rotational error coincides with the errors in the early phase of the movement. The maximum absolute rotational errors presented in Figure 8 (*Ts*,*Te*) confirm that the subjects can adapt their motor control to the unknown disturbance force field applied to their movement. The errors decreased significantly during bimanual training following bimanual baseline phase (*p *< 0.001, groups BS and BA), bimanual training following unimanual baseline phase (*p *< 0.001, groups US and UA), and also during unimanual training (*p *= 0.020, group U). Another indicator of the successful adaptation are the errors at catch trials during the baseline phase (Figure 8Bc) and the catch trials during evaluation phase (Figure 8Ec). The errors in both cases increase (both are significantly different from *B* or *Ee* with *p *< 0.001) compared to trials without (*B*) or with disturbance torque (*Ee*). In Figure 7, it is seen that the errors during baseline catch trials are mainly negative (in the direction of the *τ*_*dist*_), while the errors in the evaluation phase catch trials have the opposite direction (opposite direction of the *τ*_*dist*_). The effects of motor learning are represented in the feedforward component of the torque compensation revealed by the maximum errors. These results coincide with previous studies that showed significant learning of skills during unimanual and bimanual reaching exercises performed in a velocity-depended force field [1,28].

The second goal of the study is the analysis of transfer of skills learned during bimanual training towards single limb performance. Figure 8 (*Ts*,*Te*) presents the effects of training for all groups following the baseline phase. After the training phase with the disturbance torque active, the errors at the start of the evaluation phase (*Es*) remain the same for groups BS, BA and U. This is expected since the task basically remains the same. Groups BS and BA performed training and evaluation bimanually, while group U performed all phases using a single arm. But when the subjects in groups US and UA switched from bimanual training (*Te*) to unimanual evaluation (*Es*) their errors significantly increased. The errors at the end of the evaluation phase (*Ee*), did not further decrease compared to the start of the evaluation phase (*Es*). This means that during the evaluation phase, in spite of being similar to the unimanual training phase, no further learning during single limb performance occurred – the minimum error for unimanual performance was already reached at the end of the bimanual training phase. The performance of bimanual movements at the end of the evaluation phase (groups BS and BA) resulted in significantly lower errors compared to unimanual movements (groups US, UA and U) (*p *= 0.008).

Figure 9 shows the maximal errors for groups US, UA and U during different parts of the session. All three groups performed the baseline phase (*B*) using only their dominant arm. As expected, maximal errors for all three groups during the baseline phase are not significantly different. Groups US and UA then performed the training phase bimanually, followed again by unimanual movements of the evaluation phase. Group U used only one arm for both remaining phases (training and evaluation phase). At the start of the unimanual training phase (*Ts*) with active disturbance, the errors for group U significantly increased (*p *< 0.001). After the training phase, the error significantly (*p *= 0.032) decreased (start of the evaluation phase). This was also the final level of learned movements since the errors remained the same even at the end of the evaluation phase (*Ee*). This represents the motor learning during unimanual limb training. If these errors after unimanual training (group U) are compared to errors during unimanual evaluation (*Es* and *Ee*) after bimanual training (groups US and UA) no significant difference can be noticed. This means the bimanual training had very similar effects on unimanual skill learning as unimanual training did. The maximal rotational errors indicate a transfer of learned motor skills from bimanual training to unimanual performance. As the required forces and torques for each hand were the same in bimanual and unimanual trials, the strategies used to compensate the disturbance torque in both conditions must be similar. It is possible that both strategies are not the same, but some relation between both compensation strategies must be present.

The previously mentioned increased error for group U during the start of unimanual training (*Ts*) in Figure 9 can also be further explained. The start of unimanual training (*Ts*) represents a new, unknown situation for the subjects of group U not used to the active disturbance torque. On the other hand, the errors for groups US and UA at the start of the unimanual evaluation phase (*Es*) did not change significantly compared to the baseline errors without disturbance torque (*B*). Before the evaluation movements, the subjects performed bimanual training and learned the effects of disturbance torque on their movements. We can compare these two cases (*Ts* for group U and *Es* for groups US and UA) as these were the first unimanual trials with disturbance torque for all three groups (previously groups US and UA performed bimanual training while group U performed only unimanual trials without the disturbance torque). Significant difference in errors of groups US (*Es*) and U (*Ts*) can be seen and also errors of groups UA (*Es*) and U (*Ts*) are significantly different. Single limb performance following bimanual training was similar to performance following unimanual training and indicates a positive transfer of skills gained during bimanual training.

One part of the study also evaluated different arm force ratios in the combined bimanual movements (50*%*:50*%* or 75*%*:25*%*). No significant difference between both groups can be noticed when bimanual groups BS and BA are compared in Figure 8. By comparing the errors of groups US and UA, we can see a small and constant difference between both groups for the training phases. The errors at the end of the session (*Ee*) for both groups are not significantly different. The progress in both groups follows a very similar pattern and none of the groups show greater skill learning. The use of different arm force ratios had an equall effect on motor learning and on unimanual performance. The skill transfer from bimanual training to unimanual performance is not affected by the arm force ratio during the bimanual training.

In Figure 10, the errors during baseline and evaluation phases with unexpected ratio changes are presented. During the baseline phase, the temporary change from asymmetric to symmetric condition (group BA) had no statistically significant influence on the rotation errors. On the other hand, the change for group BS in the opposite direction (symmetric to asymmetric) resulted in significantly increased errors (*p *< 0.001). The symmetric mode is more common and normal so the subjects adapt instantly. During the evaluation phase, the changes in rotation errors are small and significant but very similar for both groups. The change from symmetric to asymmetric condition during baseline phase had significant influence on performance, but in the opposite direction no significant differences can be seen, while during the evaluation phase small but significant error changes occurred. Previous studies also showed fast and accurate adaptation to asymmetric limb loads in healthy adults [17]. In terms of rehabilitation, the bimanual movements can be very asymmetric as the impaired arm has limited motor functions. But as the impaired arm regains functions, the bimanual movements become progressively more symmetric. The arm force ratio changes in rehabilitation happen slowly over a longer period, while in our study the change is sudden and unexpected. The results of our study show fast adaptation to symmetric movements. As non-impaired bimanual movement is normally symmetric, the movements learned during rehabilitation (asymmetric movements) could also affect the performance with increasing motor abilities (more symmetric movements).

Tracking errors presented in Figure 11 show subjects’ tracking performance. The disturbance torque had no influence on the tracking errors during the baseline phase. This is partially unexpected, since the subjects have to compensate for the disturbance and at the same time track the position of the reference object. However, it seems that in the baseline phase the tracking errors were already large without the disturbance torque and were not further increased as a result of disturbance. The tracking errors for all five groups significantly decreased at the end of training as a result of motor learning. In contrast to the baseline phase, the errors during the catch trials in evaluation phase significantly increased. It seems that when subjects optimized their performance with changed dynamics, the removal of disturbance torque had significantly larger influence on tracking performance. This is another indication of motor learning and the transfer of skills from bimanual training to unimanual performance.

Our results indicate that trials with different arm force ratios during the bimanual movements have similar effects on motor learning, which is believed to be an important mechanism of rehabilitation [3]. We also showed that bimanual training has positive effects on both bimanual and unimanual performance in healthy subjects. In our previous study with hemiparetic subjects, we showed benefits of adaptive bimanual training on bimanual performance but also on unimanual performance [23]. The results of this study and our previous findings suggest that patients using bimanual training as part of their post-stroke rehabilitation might benefit from bimanual training regardless on the participation level (arm force ratio) of the impaired limb. Adapting the assistance to the impaired limb has the advantage that the training can be adjusted to the participant’s individual changing needs, both throughout each movement and over the course of rehabilitation [29]. At the beginning of rehabilitation, the impaired limb could apply less power to the combined bimanual movement. But as it would regain more function, it would also support a greater load of the bimanual movement, ideally until it could share equal loads as the unimpaired limb – transition from asymmetric towards symmetric arm force ratio. The training could be fully adapted to the abilities of each individual patient as she/he regains specific motor functions.

## Conclusion

The paper presents the effects of training with a novel bimanual robotic system. When introduced to an unknown disturbance torque that changes the dynamics of the movements, the subjects could adapt their control scheme. Performance after the training period reached levels similar to those during movements without the disturbance. Subjects successfully learned similar skills during unimanual and bimanual training.

Bimanual training did not only improve the performance of bimanual movements, it also improved single limb performance with the dominant arm. The effects of bimanual training on single limb performance were completely comparable to the effects of unimanual training on unimanual performance. Positive transfer of skills from bimanual training to dominant limb performance was observed in both symmetric and asymmetric arm force ratio conditions during bimanual training.

Subjects divided into different groups performed movements with different arm force ratios. Tracking errors increased when the arm force ratio unexpectedly changed from symmetric to asymmetric while only small changes were spotted when the conditions changed from asymmetric to symmetric arm force ratio. Most of the bimanual movements that humans make are symmetrical. This is clearly shown since the subjects adapted fast when the condition changed from asymmetric to symmetric. As bimanual movements also shift from asymmetric to symmetric during rehabilitation, the asymmetric training could be adequate for post-stroke rehabilitation.

We showed our system to be suitable for motor learning experiments during unimanual and bimanual movements. The transfer of skills from bimanual training to single limb performance was indicated. As motor learning is believed to be an important mechanism of rehabilitation, these findings might also be incorporated into future post-stroke rehabilitation systems.

## Competing interests

The authors declare that they have no competing interests.

## Authors’ contributions

The overall design of the experiments was agreed by MT and MMi. MT and MMi developed the hardware, all related programs and implemented the study. MT carried out the experiments. MT and MMi analyzed the data and drafted the manuscript. MMu aided in the study design and in drafting the manuscript. All authors read and approved the manuscript.
